# Exercise Field Testing in Children: A New Approach for Age-Appropriate Evaluation of Cardiopulmonary Function

**DOI:** 10.1007/s00246-020-02359-2

**Published:** 2020-05-09

**Authors:** Isabelle Schöffl, Benedikt Ehrlich, Simon Stanger, Kathrin Rottermann, Sven Dittrich, Volker Schöffl

**Affiliations:** 1 Department of Pediatric Cardiology, University Hospital Erlangen-Nuremberg, 91054 Erlangen, Germany; 2 Section of Sportsmedicine and Sports Orthopaedics, Department of Orthopedic and Trauma Surgery, Klinikum, Bamberg, Germany; 3 School of Clinical and Applied Sciences, Leeds Beckett University, Leeds, Great Britain; 4 Department of Pediatrics, Klinikum, Bamberg, Germany

**Keywords:** Mobile spiroergometry, Pediatric cardiology, Cardiopulmonary function in children, Outdoor testing, VO_2max_

## Abstract

Based on the wide range of problems to effectively perform cardiopulmonary testing in young children, this study strives to develop a new cardiopulmonary exercise test for children using a mobile testing device worn in a backpack in order to test children during their natural movement habits, namely, running outdoors. A standard cardiopulmonary exercise ramp test on a cycle ergometer was performed by a group of twenty 7–10-year-old children. The results were compared with a self-paced incremental running test performed using a mobile cardiopulmonary exercise measuring device in an outdoor park. The children were able to reach significantly higher values for most of the cardiopulmonary exercise variables during the outdoor test and higher. Whereas a plateau in ${\dot{\text{V}}\text{O}}_{{2}}$ was reached by 25% of the children during the outdoor test, only 75% were able to reach a reasonable VT2, let alone ${\dot{\text{V}}\text{O}}_{{{\text{2peak}}}}$, during the bicycle test. The heart rate at VT1, the O_2_-pulse, and the OUES were comparable between both tests. OUES was also positively correlated with ${\dot{\text{V}}\text{O}}_{{{\text{2peak}}}}$ in both tests. Testing children outdoors using a mobile cardiopulmonary exercise unit represents an alternative to standard exercise testing, but without the added problems of exercise equipment like treadmills or bicycles. It allows for individualized exercise testing with the aim of standardized testing durations instead of standardized testing protocols. The running speeds determined during the outdoor tests may then be used to develop age-adapted testing protocols for treadmill testing.

## Introduction

Cardiopulmonary exercise testing (CPET) is a commonly performed, non-invasive method to evaluate cardiac symptoms and assess functional capacity in children [1] and can be considered as safe [2].

Cardiorespiratory fitness (CRF) is the most important marker of health among the health-related physical fitness components in children and adolescents [3, 4]. There is an inverse association between levels of CRF during childhood and cardiovascular disease risk factors later in life [5]. So far, there is little evidence if this holds true for preschool children even though CRF is probably just as important for health parameters in preschoolers as it is in older children [6].

This is a consequence of the fact that only few studies have reported undertaking cardiopulmonary exercise testing in children less than 8 years of age [7–11]. There are two main methods for maximal exercise testing in children and adolescents: the treadmill (TM) and the bicycle test (CE). Each of these test methods has their advantages and disadvantages. Children report consistently that they are more comfortable on the CE than on the TM [11]. Electrocardiogram and blood pressure are easier to assess and of better quality using CE [12]. On the other hand children have relatively undeveloped knee extensors. Therefore, treadmill testing is preferred over cycle ergometry in young children [9], as they lack the strength in their leg muscles to reach maximal exercise capacity. However, if optimal cycle testing conditions for each child are provided, testing at an age of 5–6 years has proven to be feasible and maximal exercise effort can be achieved [11]. This kind of testing requires age-appropriate bicycles not always available in a normal cardiopulmonary exercise laboratory [11].

Treadmill testing has the advantage of extensive clinical use and is a well-established mode of exercise testing in a pediatric setting [13]. In addition it yields the highest HR [11] and ${\dot{\text{V}}\text{O}}_{{2}}$ [11, 14, 15] due to upright posture and larger exercising muscle mass than CE [13]. Still, TM testing in young children is also difficult to achieve. One of the reasons for that difficulty is that younger and smaller children tend not to run or stop prematurely during the test [7]. Classical TM protocols use a variety of inclinations as well as speed variations to increase exercise intensity during tests [13], which is difficult for young children. Thus, several authors have allowed for the holding of a handrail in order to increase the feasibility of the test [9]. Rail holding, however, is known to increase endurance time and reduce physiological strain (e.g., HR, ${\dot{\text{V}}\text{O}}_{{2}}$) during submaximal exercise [9, 16]. The widest used TM protocol is the Bruce protocol, in which stepwise increases between stages are large and the stages long with 3 min per stage [17]. For children, the work increments may be too great, resulting in the tendency for participants to quit prematurely during the first seconds of a new stage [18]. Furthermore, the long 3 min stages may lead to perceived boredom and the steep gradient may lead to premature peripheral fatigue, ending tests without achieving maximal performance values [13].

As a consequence to the abovementioned limitations of TM and CE testing on preschool children several authors have therefore aimed at developing field-based tests to indirectly estimate the ${\dot{\text{V}}\text{O}}_{{{\text{2peak}}}}$ in this age group [6, 19], like the adapted 20 m Shuttle run test for preschool children. However, a recent review on the topic of the 20mSRT has pointed out that the test does not represent an accurate estimate of peak ${\dot{\text{V}}\text{O}}_{{2}}$ [20].

With the evolvement of mobile cardiopulmonary exercise testing accurate measurements of ${\dot{\text{V}}\text{O}}_{{{\text{2peak}}}}$ can now be achieved. Our aim was therefore to compare cardiopulmonary exercise variables measured during a maximal cycle test with a field-based running test in an outdoor park using a mobile exercise testing device. The goal was to determine natural running speeds for children in a field-based exercise test to exhaustion, possibly allowing the measurement of the respective speeds for implementing an age-appropriate TM protocol without using grade increments and without the falsifying use of handrails.

## Material and Methods

Twenty healthy children aged between seven and ten years of age agreed to participate in this study. All study participants as well as their legal guardians gave informed consent to participate in the study and for their data to be used for scientific analysis. The study as well as the parent and age-adapted children consent forms were approved by the Ethics Committee of the University of Erlangen-Nuremberg, FRG (35_18B). All participants were Caucasian, non-obese, and healthy. None took medications. The subjects were recruited from local schools. There was no attempt to recruit subjects who were particularly active.

Height and weight were measured using a stadiometer and electronic scale (Seca 704 S, Hamburg, Germany). Body composition was evaluated using a bioimpedance measuring device (BIA 101, Akern, Florence, Italy).

The parents were asked about school transport habits (walking, cycling, bus). Especially cycling to and from school has proven to positively influence cardiorespiratory fitness in school children [21, 22], whereas walking has a weaker effect [21]. Sports participation was classified into low (only physical education classes), moderate (gymnastic lessons and participation in organized sports up to 2 h weekly) or high (gymnastic lessons and more than 2 h of organized sports participation weekly) according to a questionnaire proposed by van der Cammen-van Ziip et al. [9]. Physical activity is associated with a higher cardiorespiratory fitness [23].

A small, low-dead-space respiratory valve (88 ml) with a pediatric mouthpiece and headgear was used. Gas exchange was measured continuously during each test using a breath-by-breath method and averaged over 15 s intervals. The criteria for completion of peak ${\dot{\text{V}}\text{O}}_{{2}}$ was attaining a heart rate of 195 beats/minute, determined as the most robust secondary criterion to verify ${\dot{\text{V}}\text{O}}_{{{\text{2peak}}}}$ [24]. All children were instructed to abstain from food or carbohydrate rich drinks for the two hours leading up to the test. If a plateau of oxygen uptake [25] was observed, this phenomenon was noted.

Ventilatory thresholds VT_1_ and VT_2_ were determined using the V-slope method proposed by Wasserman et al. [26]. OUES was determined by plotting ${\dot{\text{V}}\text{O}}_{{2}}$ (ml/min) against the logarithm of ${\dot{\text{V}}\text{E}}$ (ml/min) and calculating the slope of this linear relation through single regression analysis [27].

The cycle ergometry was chosen as it represents the standard exercise testing mode in Europe. All subjects underwent the cycle test first in order to record a valid 12-lead ECG at rest and during the test (MAC 2000, GE Healthcare, Chicago US), which was not possible during the outdoor setting. All tests were undertaken in the morning. The Cyclus 2, an electronically braked cycle ergometer (RBM elektronik-automation GmbH, Leipzig, Germany) with a standard children’s mountain bike for children aged 8–10 years (MTB 20″ Racing Boy 320 blue, Decathlon) was used for the cycle test. Before each test the height of the saddle and the handlebars were adjusted to the height of each child. Subjects warmed up on the bike for a period of 5 min. After a short rest period (10 min) subjects were fitted with a heart rate (HR) monitor (Polar H7 Bluetooth Smart 4.0® heart rate sensor, Kempele, Finland). The mask was fitted. The same mobile exercise equipment was used for the CE test and for the outdoor test (Metamax®, Cortex, Leipzig, Germany). The children began by cycling at 10 W for a 2 min warm-up phase. Work rate was then continuously incremented in a linear ramp pattern with an increase of 10 W/min, a protocol comparable to one proposed by Cooper et al. [28] but with a warm-up phase of 10 W instead of 0 W. We chose to adapt the protocol as we tested healthy children. The children were instructed to maintain a constant pedaling rate above 60 rpm. Children were verbally encouraged to cycle until exhaustion. All tests were performed by the same researchers.

The outdoor test was performed a minimum of two days and maximum of two weeks after the bicycle test in order to guarantee a sufficient recovery but also to keep a comparable fitness level and anthropometry at the time of the second test. All tests were again carried out in the morning at or around the same time that each child had been tested on the CE. The climatic conditions were comparable to the cycle tests but not identical, as humidity, wind speed and even temperature cannot be controlled in an outdoor setting. The children warmed up in the same way as for the CE and then fitted with the equipment as described before. The test was designed as a self- or researcher-paced incremental test rather than a ramp or true step test. Each child was instructed prior to the test. The first increment would consist of walking at a leisurely pace. After a period of 2 min the speed would be increased to an easy jog (2 min), then to running with some effort (2 min). This speed was described as a speed they could be able to maintain for a longer period but should feel tiring. Lastly they would have to try to run as fast as possible for as long as possible, so as if chasing another child who could take away their prize. An experienced researcher and running coach for children performed the whole test alongside each child controlling the speed and the time of each step using a watch equipped with a GPS sensor (Garmin Fenix 5S, Garmin, Olathe, USA) in order to slow down or motivate the respective child if need be. All tests were accompanied by the same team. The speed was adapted to the capacities of each child by observation of as well as feedback from the child. A GPS sensor integrated in the mobile unit recorded the speed of the child at every moment of the test. Running was performed on a flat, wide and even trail over the whole distance of the test.

Statistical analysis was performed using Microsoft Excel 2000® for data collection and SPSS 12.0® (SPSS Inc., Chicago, IL). All measured values are reported as means and standard deviations. The Kologomorov-Smirnov test was used to check for normal distribution. Homogeneity of variance was investigated using Levine’s F-test. For normally distributed variables differences between the two test protocols were assessed with paired t-tests, otherwise the Wilcoxon or the Whitney–Mann U tests were used. All tests were 2 tailed, a 5% probability level was considered significant.

Pearson or Spearmen correlation coefficients were used to investigate univariate correlation between independent variables and ${\dot{\text{V}}\text{O}}_{{{\text{2peak}}}}$.

## Results

We were able to test 20 children on the CE but only 19 outdoors as one girl caught a viral infection between the two tests. Out of the 20 children 12 were boys and 8 were girls. Their age, anthropometric data, as well as their sports participation and their school transport habits are represented in Table 1. The boys and girls did not differ significantly from each other with respect to the anthropometric variables. The collective recruited in our study showed a high percentage of physical activity, but mainly walked to school. There were no correlations between school transport habits and the ${\dot{\text{V}}\text{O}}_{{{\text{2peak}}}}$. Nor was there a discernable difference between school transport habits and the respective tests. Children using the bicycle to get to school did not show a higher cardiorespiratory fitness during the bicycle test than during the outdoor test. The same observations were true for the amount of physical activity which did not statistically impact on the ${\dot{\text{V}}\text{O}}_{{{\text{2peak}}}}$ or other cardiorespiratory measurements.

**Table 1:** Anthropometric data as well as sports participation and school transport habits as means and standard deviation

	Girls (*n* = 8)	Boys (*n* = 12)
Age (years)	7.8 (0.3)	8.1 (0.3)
Height	128.4 (1.5)	132.0 (1.7)
Weight	25.7 (1.4)	27.8 (1.7)
BMI	15.5 (0.6)	15.8 (0.6)
BSA (m^2^)	1.02 (0.13)	0.97 (0.09)
Fat Free Mass (FFM)	21.3 (1.2)	23.1 (1.0)
Sports participation	62% with > 2 h/week 38% with 0–2 h/week	100% with > 2 h/week
School transport habits	50% walk 38% bike	83% walk 8% bike 11% bus

Both tests were well tolerated by the children. The main reason for stopping the CE test was weakness in the legs and most outdoor tests were ended when the speed during the last step could not further be increased and children reported being exhausted.

During the CE 15 (75%) children reached the second ventilatory threshold (VT2), whereas during the outdoor test all children reached VT2 and a realistic ${\dot{\text{V}}\text{O}}_{{{\text{2peak}}}}$. A plateau in ${\dot{\text{V}}\text{O}}_{{{\text{2peak}}}}$ was reached by 4 (21%) children during the outdoor test and none during the cycle test. An R value of > 1.0 was observed for all children during the outdoor tests and 90% during the CE tests. A HR value of > 95% of the age-predicted maximum [29] was reached by 81% of the children during the outdoor test and by 15% during the CE test.

**Fig. 1:**
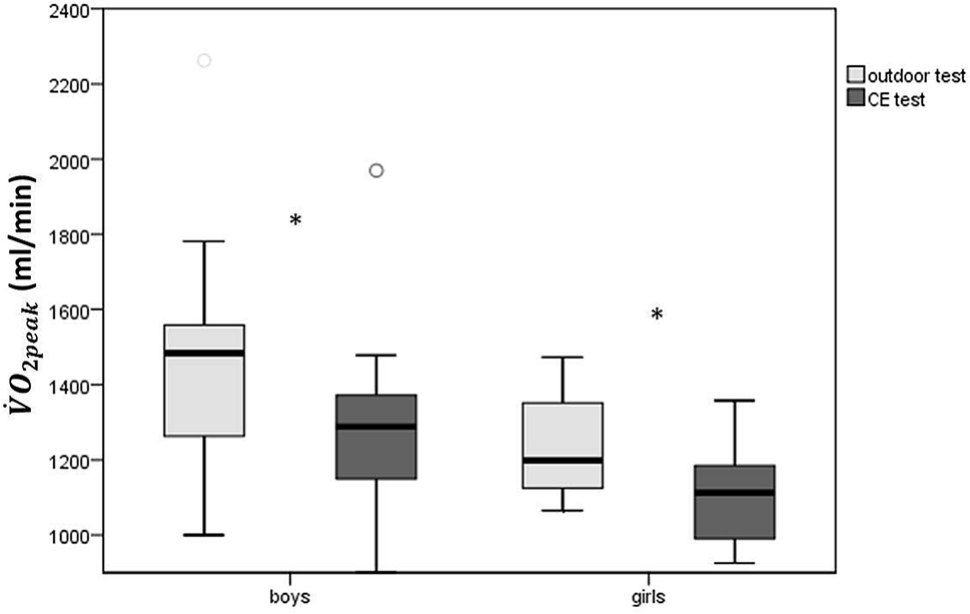
Mean and standard deviation of ${\dot{\text{V}}\text{O}}_{{{\text{2peak}}}}$ during the outdoor and the CE tests for boys and girls. There was a significant difference between the two tests for boys and girls, but no significant differences between the two genders for each test

The main parameters studied during the CE and the outdoor tests are represented in Table 2. Almost all parameters showed a significant difference between the two tests, yielding higher values for the outdoor test than for the CE. The measured ${\dot{\text{V}}\text{O}}_{{{\text{2peak}}}}$ was 15% higher during the outdoor test (s. Figure 1). The only exceptions were the O_2_ pulse (${\dot{\text{V}}\text{O}}_{{2}}$/Heart Rate), the heart rate at VT1 and VT2, the peak respiratory exchange ratio (RER), and the oxygen uptake efficiency slope (OUES).

**Table 2:** Means and standard deviations of the cardiopulmonary exercise test parameters on the bicycle versus the outdoor test with the respective *p *values for student *t *test between two tests (*depicts a significant difference between the two tests)

	Cycle test		Outdoor test		*P *value
	Boys (*n* = 12)	Girls (*n* = 8)	Boys (*n* = 12)	Girls (*n* = 7)	
${\dot{\text{V}}\text{O}}_{{{\text{2peak}}}}$ (ml/min)*	1304.17 (259.1)	1106.8 (157.5)	1470.0 (333.0)	1241.6 (159.3)	0.000
${\dot{\text{V}}\text{O}}_{{{\text{2peak}}}}$ (ml/min/kg)*	47.3 (5.0)	44.7 (6.4)	52.8 (2.5)	50.0 (4.8)	0.000
${\dot{\text{V}}\text{O}}_{{2}}$ at VT1 (ml/kg/min)*	26.1 (5.9)	27.3 (4.1)	23.6 (3.3)	21.6 (5.7)	0.022
${\dot{\text{V}}\text{O}}_{{2}}$ at VT2 (ml/kg/min)	44.4 (6.7)	39.3 (5.4)	48.1 (3.1)	43.1 (4.8)	0.005
Peak Work rate (Watt/kg) and peak speed (km/h)	3.0 (0.9)	2.9 (0.6)	11.4 (1.2)	12.5 (1.5)	
Peak heart rate (bpm)*	180.6 (6.8)	183.9 (7.0)	192.5 (6.4)	199.3 (8.1)	0.000
Heart rate at VT1 (bpm)	136 (16)	119 (25)	132 (14)	131 (7)	0.732
Peak O_2_ pulse (ml/heart beat)	7.5 (1.4)	6.1 (1.1)	7.7 (1.6)	6.4 (0.8)	0.163
Peak RER	1,16 (0,13)	1,16 (0,15)	1,19 (0,7)	1,25 (0,05)	0.066
Peak ${\dot{\text{V}}\text{E}}$ (ml/min)*	49.6 (10.6)	42.7 (5.5)	58.6 (11.5)	52.5 (9.1)	0.000
OUES (oxygen uptake efficiency slope)*	1.6 (0.4)	1.4 (0.2)	1.9 (0.5)	1.4 (0.2)	0.061
Exercise time (min)	7.5 (1.8)	7.0 (0.8)	7.1 (0.5)	6.9 (0.8)	0.501

There was a significant correlation between OUES and ${\dot{\text{V}}\text{O}}_{{{\text{2peak}}}}$ in the outdoor setting as well as during the CE tests.

Boys and girls achieved comparable results during both test modalities. There were no significant differences with respect to all CPET measurements.

**Fig. 2:**
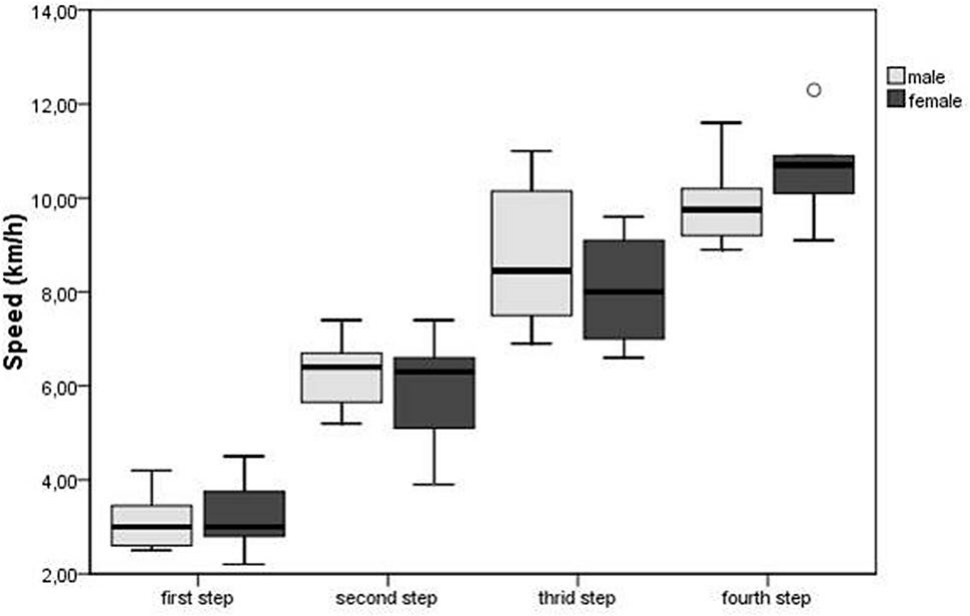
Speeds recorded at each step during the outdoor test with means and standard deviation

Figure 2 depicts the median speeds of the four increments during the outdoor test. The overall (girls and boys taken together) mean speed for the first step was 3.16 km/h (sd 0.69 km/h), the second step was 6.12 km/h (sd 0.9 km/h), the third step was 8.5 km/h (sd 1.42 km/h) and the last step was 10.11 km/h (sd 0.9 km/h).

## Discussion

Exercise testing is a valuable tool in the assessment of healthy children and those with a chronic cardiopulmonary disease. However, especially for testing small children, the use of classical treadmill and bicycle protocols is problematic [18]. There are several points in favor of both methods of exercise testing. The main disadvantage in our view is the fact that children have underdeveloped knee extensor muscles. These are the muscle groups most needed in a bicycle test as well as in classical treadmill tests where an increase in gradient is used for increasing the work load [8, 9, 17]. Furthermore, running on a treadmill involves a certain degree of motor skills which are not present at a young age [18]. In our experience, even when safety precautions such as using a tether are implemented, young children still struggle with walking and running on treadmills, even though they are perfectly capable of walking and running in normal life.

The aim to use field-based tests for evaluating the cardiopulmonary capacity in children has been widely used but lacks validation [30]. We therefore wanted to validate a field-based incremental exercise test using a mobile cardiopulmonary exercise test unit in order to implement a safe, new testing method which may then be applied to smaller children or cardiopulmonary limited children.

Previous studies comparing TM with CE protocols [11, 14, 15, 31] have found differences of 5–10% for ${\dot{\text{V}}\text{O}}_{{{\text{2peak}}}}$ between the two test methods. We compared a CE test with an outdoor running test because CE testing accounts for the most common CPET performed in Europe and is the method children feel most comfortable with. We preferred to account for the established differences between the two test methods, as the main goal of the study was to establish the feasibility of this new testing method.

In our study, cardiorespiratory values were also higher during the outdoor test than during the CE test. The absolute values for ${\dot{\text{V}}\text{O}}_{{{\text{2peak}}}}$ in our study differed by 13%. As the outdoor test can best be compared to a treadmill running protocol these results reflect what the earlier studies established. We also recorded significantly higher values for the outdoor tests in comparison with the CE tests with respect to HR, ${\dot{\text{V}}\text{O}}_{{2}}$ at VT2, and ${\dot{\text{V}}\text{E}}_{\max }$_,_ which is mostly in accordance with the present literature. Interestingly, the peak RER registered in our study was also significantly higher during the outdoor protocol than on the bicycle. So far it is believed that higher RER values are recorded using cycle protocols [11, 14] because of the influence of local muscle fatigue, which is more pronounced in young children than in adults [11]. In our study, all subjects had to stop the CE test due to local muscle fatigue, whereas they stated cardiopulmonary exhaustion as the cause for terminating the outdoor test. This is also reflected in the fact that we were able to discern a plateau in ${\dot{\text{V}}\text{O}}_{{2}}$ in 21% of the cases during the outdoor tests, while none achieved this during the CE tests. On the contrary 10% could not even reach an RER value of greater than 1, and 25% did not reach the second ventilatory threshold during the CE tests due to muscle fatigue of the thigh.

With regards to the absolute cardiopulmonary exercise variables reached in our collective of healthy children between the ages of 7–10 years, it was obvious that they achieved similar results as previously reported for CE tests, namely 47 ml/kg/min for boys and 44.7 ml/kg/min for girls [11, 28, 32, 33] but reached superior values during the outdoor tests, namely 52.8 ml/kg/min for boys and 50 ml/kg/min for girls, when compared with younger children [11] performing a treadmill test which incorporated an increase in inclination. The same was true when compared to older children performing a comparable test profile [15]. However, they achieved comparable results when compared with older children performing a new treadmill protocol which only increased in speed and did not incorporate an increase in inclination [13]. These observations imply that testing children with an increase in gradient may not be suitable as it does not adequately reflect their familiar movement patterns. It is far more natural for children to increase the speed at which they run in order to reach a maximum of effort than walking up steep hills. The reason for using TM protocols which include an increase in inclination is the relative risk for younger children on a treadmill. As the speed can be kept lower when increasing the inclination, the risk is considered less [9]. However, as this yields lower results than when testing using a flat surface, we favor a protocol which involves running with increasing speed.

Several cardiopulmonary parameters were not significantly different between the two tests, namely the heart rate at VT1 and the ${\dot{\text{V}}\text{E}}$ at VT1. These results reflect that the ventilatory threshold can be accurately determined with both tests if the children are able to reach a ${\dot{\text{V}}\text{O}}_{{{\text{2peak}}}}$ or at least an RER > 1 with comparable results. In short, both tests can be used for providing exercise recommendations in healthy children between the ages of 7 and 10 years. However, the applicability of a CE test for smaller children remains questionable if not altogether impossible if the children are too small for the bicycle or are not used to this exercise yet [18].

There was also no significant difference between the exercise times required to reach exhaustion in both tests. With a medium exercise test length of 7 min the exercise time was comparably short [13], but we still believe this time to be sufficient, as all subjects performing mobile testing, reached a clearly discernible ${\dot{\text{V}}\text{O}}_{{{\text{2peak}}}}$ and ventilatory thresholds could be determined for every test. Since boredom due to long stages and consequently long exercise time can lead to premature ending of the test before reaching a reasonable cardiopulmonary exertion [13], an exercise time of 6–8 min seems a reasonable compromise. There is much discussion about standardizing cardiopulmonary exercise test protocols for better comparability [8] and for permitting long-term observation. However, the maturation of children entails that using the same test protocol for children of increasing age leads to ever longer exercise tests, which will in and of itself lead to a bias in the results. When including children with cardiac or pulmonary disease the exercise time will most likely be much shorter as they reach cardiopulmonary exertion much sooner than their healthy peers. Our approach allows for the children to run at their own pace, reaching cardiopulmonary exertion after duration of 6–8 min. As they mature this exercise time remains constant but the speed for each step will most likely increase, allowing for cardiopulmonary exertion in a comparable time frame.

The correlation between OUES and ${\dot{\text{V}}\text{O}}_{{{\text{2peak}}}}$ were significant for both tests. The values were well within data recorded in literature for that age group [34]. Several studies have investigated the use of OUES as a valid submaximal alternative for ${\dot{\text{V}}\text{O}}_{{{\text{2peak}}}}$ estimation in children [27, 34, 35]. As the OUES is being determined during the last part of the CPET no maximal effort is required but VT2 needs to be reached. As we were able to record a significant correlation between OUES and ${\dot{\text{V}}\text{O}}_{{{\text{2peak}}}}$ this could represent an alternative parameter when ${\dot{\text{V}}\text{O}}_{{{\text{2peak}}}}$ cannot be achieved.

The fact that the boys had a higher, if not significantly so, ${\dot{\text{V}}\text{O}}_{{{\text{2peak}}}}$ than the girls in our study reflects the current literature [36, 37]. Interestingly, even though the ${\dot{\text{V}}\text{O}}_{{{\text{2peak}}}}$ was lower in the group of girls in this study, the girls ran faster during the outdoor test than the boys. Again, this difference did not reach significance. However, there is no question about the ability of girls to perform to a comparable level as the boys at 7–10 years of age.

The new testing method has several limitations that need to be taken into consideration. Bad weather conditions can impede testing in the park or on a track, making indoor testing necessary. Weather conditions and environmental conditions may also influence the measured results as the standard environmental settings of a laboratory are left behind. Calibration of the test material in the outdoor setting previous to each test battery is therefore crucial for limiting these effects on the test results. Special mobile exercise testing equipment with incorporated GPS sensors and Heart rate monitors are necessary for being able to perform these tests, so far limiting their applicability in most clinical settings. In this study the heart rate was recorded using a standard Polar heart rate monitor. This does not allow for continuous ECG monitoring and thus limits the applicability of the testing method for routine clinical use. However, Custo med has recently developed a 3-channel ECG integrated into a belt (Custo Guard, Custo, Germany) similar to the standard heart rate monitors used during physical activity. Since our study has been conducted, Cortex Germany has integrated Custo Guard into their software so that a 3-channel ECG can now be recorded even during outdoor testing. So far this test has only been carried out in a group of healthy children. When implementing this kind of test in a patient group typical for pediatric cardiology like patients with Fontan circulation, or patients with cardiomyopathy, the test needs to be adapted to their cardiorespiratory fitness with regards to the achievable speeds in each step. More experience and more studies are therefore needed to implement this test in a patient group. However, when accompanying the children during the test, the visual and verbal feedback from the children to the medical supervisor allowed for good adaptation to the respective speeds achievable by each child. We believe that after acquiring some experience with this kind of outdoor test, conducting the same test in a group of patients with heart disease is feasible when special attention is paid to the visual and verbal feedback provided by each patient. Of course when testing patients, a certain backup of medical equipment is crucial. During our study we had an AED (automatic external defibrillator) and backpack with emergency equipment on site. However, patients need to be tested in close proximity to a care facility so that in a case of emergency all necessary equipment is readily available.

As the children ran up to 12 km/h during the last step of the test, the medical supervisor needs to exhibit a certain degree of fitness in order to keep up with the children.

Incremental exercise testing of children between 7 and 10 years of age in the park using a mobile cardiopulmonary exercise testing unit allows for accurate determination of ${\dot{\text{V}}\text{O}}_{{{\text{2peak}}}}$. As the children can influence the speed at which they complete each step, the speed can be adapted to their capacities, allowing for exercise testing of even smaller children or children with cardiopulmonary limitations.

## References

[1] Paridon SM, Alpert BS, Boas SR, Cabrera ME, Caldarera LL, Daniels SR, Kimball TR, Knilans TK, Nixon PA, Rhodes J, Yetman AT, American Heart Association Council on Cardiovascular Disease in the Young CoAH, Obesity in Y (2006). Clinical stress testing in the pediatric age group: a statement from the American Heart Association Council on Cardiovascular Disease in the Young, Committee on Atherosclerosis, Hypertension, and Obesity in Youth. Circulation.

[2] Ghosh RM, Gates GJ, Walsh CA, Schiller MS, Pass RH, Ceresnak SR (2015). The prevalence of arrhythmias, predictors for arrhythmias, and safety of exercise stress testing in children. Pediatr Cardiol.

[3] Ortega FB, Ruiz JR, Castillo MJ, Sjöström M (2007). Physical fitness in childhood and adolescence: a powerful marker of health. Int J Obes.

[4] Ruiz JR, Castro-Pinero J, Artero EG, Ortega FB, Sjostrom M, Suni J, Castillo MJ (2009). Predictive validity of health-related fitness in youth: a systematic review. Br J Sports Med.

[5] Nordström A, Högström G, Nordström P (2014). High aerobic fitness in late adolescence is associated with a reduced risk of myocardial infarction later in life: a nationwide cohort study in men. Eur Heart J.

[6] Mora-Gonzalez J, Cadenas-Sanchez C, Martinez-Tellez B, Sanchez-Delgado G, Ruiz JR, Léger L, Ortega FB (2017). Estimating VO2max in children aged 5–6 years through the preschool-adapted 20-m shuttle-run test (PREFIT). Eur J Appl Physiol.

[7] Eiberg S, Hasselstrom H, Grønfeldt V, Froberg K, Svensson J, Andersen LB (2005). Maximum oxygen uptake and objectively measured physical activity in Danish children 6–7 years of age: the Copenhagen school child intervention study. Br J Sports Med.

[8] Dubowy K-O, Baden W, Bernitzki S, Peters B (2008). A practical and transferable new protocol for treadmill testing of children and adults*. Cardiol Young.

[9] van der Cammen-van Zijp MHM, IJsselstijn H, Takken T, Willemsen SP, Tibboel D, Stam HJ, van den Berg-Emons RJG (2009). Exercise testing of pre-school children using the Bruce treadmill protocol: new reference values. Eur J Appl Physiol.

[10] Wessel HU, Strasburger JF, Mitchell BM (2001). New standards for the bruce treadmill protocol in children and adolescents. Pediatr Exerc Sci.

[11] LeMura LM, von Duvillard SP, Cohen SL, Root CJ, Chelland SA, Andreacci J, Hoover J, Weatherford J (2001). Treadmill and cycle ergometry testing in 5- to 6-year-old children. Eur J Appl Physiol.

[12] Takken T, Bongers BC, Mv B, Haapala EA, Hulzebos EHJ (2017). Cardiopulmonary exercise testing in pediatrics. Ann Am Thorac Soc.

[13] Duff DK, De Souza AM, Human DG, Potts JE, Harris KC (2017). A novel treadmill protocol for exercise testing in children: the British Columbia Children's Hospital protocol. BMJ Open Sport Exerc Med.

[14] Boileau RA, Bonen A, Heyward VH, Massey BH (1977). Maximal aerobic capacity on the treadmill and bicycle ergometer of boys 11–14 years of age. J Sports Med Phys Fit.

[15] Armstrong N, Williams J, Balding J, Gentle P, Kirby B (1991). The peak oxygen uptake of British children with reference to age, sex and sexual maturity. Eur J Appl Physiol Occup Physiol.

[16] Van Der Cammen-van Zijp MHM, Van Den Berg-Emons RJG, Willemsen SP, Stam HJ, Tibboel D, IJsselstijn H (2010). Exercise capacity in Dutch children: new reference values for the Bruce treadmill protocol. Scand J Med Sci Sports.

[17] Bruce RA, Blackmon JR, Jones JW, Strait G (1963). Exercising testing in adult normal subjects and cardiac patients. Pediatrics.

[18] Hebestreit H (2004). Exercise testing in children—what works, what doesn't, and where to go?. Paediatr Respir Rev.

[19] Leger LA, Mercier D, Gadoury C, Lambert J (1988). The multistage 20 metre shuttle run test for aerobic fitness. J Sports Sci.

[20] Tomkinson GR, Lang JJ, Blanchard J, Leger LA, Tremblay MS (2019). The 20-m shuttle run: assessment and interpretation of data in relation to youth aerobic fitness and health. Pediatr Exerc Sci.

[21] Larouche R, Saunders TJ, Faulkner G, Colley R, Tremblay M (2014). Associations between active school transport and physical activity, body composition, and cardiovascular fitness: a systematic review of 68 studies. J Phys Act Health.

[22] Ramirez-Velez R, Garcia-Hermoso A, Agostinis-Sobrinho C, Mota J, Santos R, Correa-Bautista JE, Amaya-Tambo DC, Villa-Gonzalez E (2017). Cycling to school and body composition, physical fitness, and metabolic syndrome in children and adolescents. J Pediatr.

[23] Grgic J, Dumuid D, Bengoechea EG, Shrestha N, Bauman A, Olds T, Pedisic Z (2018). Health outcomes associated with reallocations of time between sleep, sedentary behaviour, and physical activity: a systematic scoping review of isotemporal substitution studies. Int J Behav Nutr Phys Act.

[24] Barker AR, Williams CA, Jones AM, Armstrong N (2011). Establishing maximal oxygen uptake in young people during a ramp cycle test to exhaustion. Br J Sports Med.

[25] Massin MM (2014). The role of exercise testing in pediatric cardiology. Arch Cardiovasc Dis.

[26] Beaver WL, Wasserman K, Whipp BJ (1986). A new method for detecting anaerobic threshold by gas exchange. J Appl Physiol.

[27] Akkerman M, van Brussel M, Bongers BC, Hulzebos EH, Helders PJ, Takken T (2010). Oxygen uptake efficiency slope in healthy children. Pediatr Exerc Sci.

[28] Cooper DM, Weiler-Ravell D, Whipp BJ, Wasserman K (1984). Aerobic parameters of exercise as a function of body size during growth in children. J Appl Physiol.

[29] Gelbart M, Ziv-Baran T, Williams CA, Yarom Y, Dubnov-Raz G (2017). Prediction of maximal heart rate in children and adolescents. Clin J Sport Med.

[30] Tomkinson GR, Lang JJ, Blanchard J, Léger LA, Tremblay MS (2019). The 20-m shuttle run: assessment and interpretation of data in relation to youth aerobic fitness and health. Pediatr Exerc Sci.

[31] Turley KR (1985). Wilmore JH (1997) Cardiovascular responses to treadmill and cycle ergometer exercise in children and adults. J Appl Physiol.

[32] Harkel ADJT, Takken T, Van Osch-Gevers M, Helbing WA (2011). Normal values for cardiopulmonary exercise testing in children. Eur J Cardiovasc Prev Rehabil.

[33] Takken T, Giardini A, Reybrouck T, Gewillig M, Hovels-Gurich HH, Longmuir PE, McCrindle BW, Paridon SM, Hager A (2012). Recommendations for physical activity, recreation sport, and exercise training in paediatric patients with congenital heart disease: a report from the Exercise, Basic & Translational Research Section of the European Association of Cardiovascular Prevention and Rehabilitation, the European Congenital Heart and Lung Exercise Group, and the Association for European Paediatric Cardiology. Eur J Prev Cardiol.

[34] Bongers BC, Hulzebos HJ, Blank AC, van Brussel M, Takken T (2011). The oxygen uptake efficiency slope in children with congenital heart disease: construct and group validity. Eur J Cardiovasc Prev Rehabil.

[35] Bongers BC, Hulzebos EH, Helbing WA, ten Harkel AD, van Brussel M, Takken T (2016). Response profiles of oxygen uptake efficiency during exercise in healthy children. Eur J Prev Cardiol.

[36] Armstrong N, Welsman JR (1994). Assessment and interpretation of aerobic fitness in children and adolescents. Exerc Sport Sci Rev.

[37] Armstrong N, Welsman J (2001). Peak oxygen uptake in relation to growth and maturation in 11- to 17–year-old humans. Eur J Appl Physiol.

